# Multiple Fan-Beam Optical Tomography: Modelling Techniques

**DOI:** 10.3390/s91108562

**Published:** 2009-10-27

**Authors:** Ruzairi Abdul Rahim, Leong Lai Chen, Chan Kok San, Mohd Hafiz Fazalul Rahiman, Pang Jon Fea

**Affiliations:** 1 Process Tomography Research Group (PROTOM), Department of Control & Instrumentation Engineering, Faculty of Electrical Engineering, Universiti Teknologi Malaysia, 81310 UTM Skudai, Johor, Malaysia; E-Mail: kschan@istone.com.my (C.K.S.); 2 Tomography Imaging Research Group, School of Mechatronic Engineering, Universiti Malaysia Perlis, 02600 Arau, Perlis, Malaysia; E-Mail: hafiz@unimap.edu.my (M.H.F.R.)

**Keywords:** optical tomography, sensor modelling

## Abstract

This paper explains in detail the solution to the forward and inverse problem faced in this research. In the forward problem section, the projection geometry and the sensor modelling are discussed. The dimensions, distributions and arrangements of the optical fibre sensors are determined based on the real hardware constructed and these are explained in the projection geometry section. The general idea in sensor modelling is to simulate an artificial environment, but with similar system properties, to predict the actual sensor values for various flow models in the hardware system. The sensitivity maps produced from the solution of the forward problems are important in reconstructing the tomographic image.

## 1. Introduction

**Figure 1.**
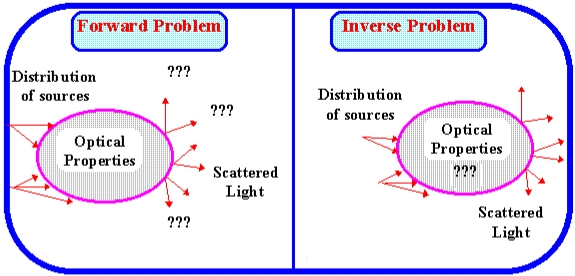
Illustration of forward problem and inverse problem.

Tomographic images are usually derived using certain image reconstruction algorithms. Reconstruction of images for an object from a set of cross-sectional projection data of the object is a unique processing problem. It is necessary to solve the forward problem first in order to derive the algorithm which provides solutions to the inverse problem. Figure 1 illustrates the forward problem and inverse problem in optical tomography.

The motivation for the sensor modelling in this research is to simulate an artificial environment of an optical tomography system with multiple fan beam projection with similar system properties to predict the actual sensor values for various flow models in the hardware system.

## 2. Sensor Modelling

In previous research, three approaches were used to determine sensor output modelling. The models are the optical path length model, optical attenuation model and optical path width model. Ruzairi [1] first applied the optical path length model in his work to predict amplitude of sensor output voltage, which resulted in a proportional relation between the output voltage amplitudes and the particle flow rate. Research by Ibrahim *et al.* [2] has shown that the optical attenuation model is suitable for use in liquid/gas systems where both media are transparent but possess different optical attenuation coefficients. Chan [3] has investigated the two previous optical models and concluded that both models are unsuitable for his project, which applies light intensity measurements and uses solid particles as flow materials. In his deduction, he concluded that the signal conditioning system that employs light intensity measurement is not suitable for the optical path length model and that solid materials used as flowing objects have high absorption characteristics which make use of the optical attenuation model unsuitable as this kind of model must assume both conveyed and conveying mediums are transparent. Thus, this research adopts the optical path width model applied by Chan *et al.* [4] where sensor modelling is done based on the width of the sensing beams within the pipeline projections. Generally, the basic principle of the sensor modelling in this project is light beams transmitting in a straight line to receivers [5]. The sensor output is dependent on the blockage effect when solid materials intercept the light beam. As the first step to approach the sensor modelling, a few assumptions are made to obtain the model. The assumptions are as follows:

- All incident lights on the surface of the solid materials are fully absorbed by the object [3].
- The effects of light diffractions and scatterings are ignored because the primary effect is the attenuation of optical energy by particles intercepting the beam [6].
- The intensity of the light emission from each infrared emitter is uniform along all covered directions. A single projection will produce six light beams from the infrared to the photodiodes.

**Figure 2.**
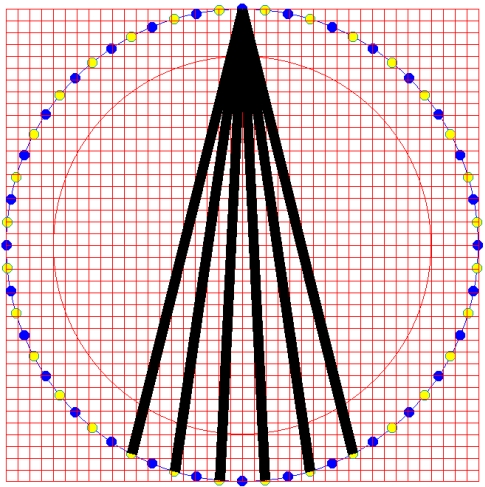
Single transmitter light emission.

According to Chan [3,7], the width of each beam is dependent on the angles of the light that arrives at the corresponding receivers. His application involves the transmitter's single emission which is received by all the receivers in the pipeline. However, in this multiple fan beam projection application using optical fibre sensors, the width of the light beam is small and the transmission angle of the transmitter is 30 degrees where only six receivers will receive light from one transmitter, as illustrated in Figure 2.

**Figure 3.**
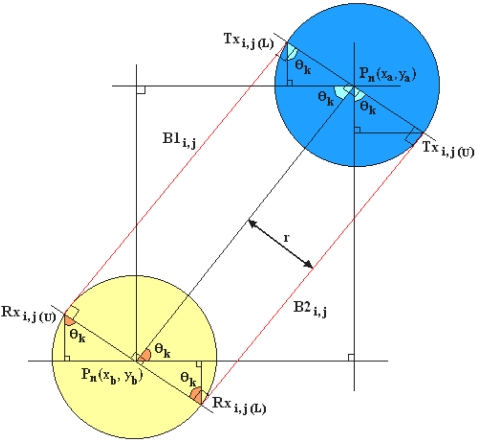
Tangent modelling of sensors.

The width of each beam is considered constant along the emission towards all the corresponding receivers by using the tangent approach. This approach is taken based on the investigation on Chan's modeling [3,7]. It is found that the width difference of light beams from one transmitter to the corresponding receivers affected by the transmitting angle of 30 degrees is very small (±4% or ±0.1mm difference). Based on this calculation and to make the modelling in Visual C++ programming easier, the tangent modelling of the sensors is as shown in Figure 3.

From Figure 3, P_n_(x, y) is the centre of the sensor's coordinates. After mapping the transmitters and receivers onto the map with a scale from (0, 0) to (640, 640), there is a need to simplify the boundary determination for each emission light from emitters to receivers [3]. B1_i, j_ and B2_i, j_ are the border lines of the light path while Tx_i, j (L)_, Rx_i, j (L)_, Rx_i, j (L)_ and Rx_i, j (L)_ are the coordinates which forms a polygon to satisfy the boundary points.

For different sets of transmitter and receiver's light beams, there are different sets of boundary points/coordinates. Firstly, θ_k_ must be calculated with Equation 1:

(1) $$\theta_{k}={\text{tan}}^{-1}\left(\frac{y_{a}-y_{b}}{x_{a}-{\text{x}}_{b}}\right)$$

where *θ_k_* = the angle needed to find boundary coordinates, *y_a_* = the y-th coordinate of the n-th transmitter, *x_a_* = the x-th coordinate of the n-th transmitter, *y_b_* = the y-th coordinate of the n-th receiver and *x_b_* = the x-th coordinate of the n-th receiver.

Once the centre coordinates, P_n_(x, y) and θ_k_ have been obtained, the boundary coordinates can be determined with Equations 2-9:

(2) $$Tx_{i,j(L)}.x=x_{a}\pm r\sin\theta_{k}$$

(3) $$Tx_{i,j(L)}.y=y_{a}\pm r\cos\theta_{k}$$

(4) $$Tx_{i,j(U)}.x=x_{a}\pm r\sin\theta_{k}$$

(5) $$Tx_{i,j(U)}.y=y_{a}\pm r\cos\theta_{k}$$

(6) $$Rx_{i,j(L)}.x=x_{b}\pm r\sin\theta_{k}$$

(7) $$Rx_{i,j(L)}.y=y_{b}\pm r\cos\theta_{k}$$

(8) $$Rx_{i,j(U)}.x=x_{b}\pm r\sin\theta_{k}$$

(9) $$Rx_{i,j(U)}.y=y_{b}\pm r\cos\theta_{k}$$

where *r* = radius of the sensor which is 7 pixels, *i, j* = the beam for i-th transmitter and j-th receiver, *Tx_i,j(L)_ x* = the x-th coordinate of the lower boundary of corresponding transmitter, *Tx_i,j(L)_ y* = the y-th coordinate of the lower boundary of corresponding transmitter, *Tx_i,j(U)_ x* = the x-th coordinate of the upper boundary of corresponding transmitter, *Tx_i,j(U)_ y* = the y-th coordinate of the upper boundary of corresponding transmitter, *Rx_i,j_*_(_*_L_*_)_·*x* = the x-th coordinate of the lower boundary of corresponding receiver, *Rx_i,j(L)_ y* = the y-th coordinate of the lower boundary of corresponding receiver, *Rx_i,j(U)_ x* = the x-th coordinate of the upper boundary of corresponding receiver and *Rx_i,j(U)_ y* = the y-th coordinate of the upper boundary of corresponding receiver.

In order to obtain a connection between the sensors and solid particles that intercepts the light beams during several flow conditions, the optical path width model is being applied. Only one modelling approach is needed to generate the sensor outputs for light interception of solid particles with different sizes and shapes. Prior to this, the upper and lower boundary points have been found. On the same image plane, the light beams for all the corresponding transmitters and receivers are being drawn. In the situation of six receivers receiving light from one transmitter, the total of light paths in one frame of light emission is 192. Each light beam is in the form of straight line, therefore there is a gradient for every light path according to the linear graph equation. The gradient here means a number that represents the steepness of a straight line. By using the boundary points, the gradients of the light paths can be obtained as referred to Equation 10:

(10) $$m_{i,j}=\frac{\left(Rx_{i,j(U)}.y\right)-\left(Tx_{i,j(L)}.y\right)}{\left(Rx_{i,j(U)}.x\right)-\left(Tx_{i,j(L)}.x\right)}$$

where *m_i,j_* = the gradient/slope of light path for i-th transmitter and j-th receiver, *Rx_i,j(U)_ y* = the y-th coordinate of the upper boundary of corresponding receiver, *Tx_i,j(L)_ y* = the y-th coordinate of the lower boundary of corresponding transmitter, *Rx_i,j(U)_ x* = the x-th coordinate of the upper boundary of corresponding receiver and *Tx_i,j(L)_ x* = the x-th coordinate of the lower boundary of corresponding transmitter.

The next step is to investigate the sensor output condition if the light path is being intercepted by solid particles. The particles might intercept the light beams in many different ways, for example fully or partially blocking the light path. By using the linear graph extrapolation method, the ‘y intercept’ of each boundary lines for all possible combination of transmitters and receivers are attained by following Equation 11 and Equation 12. Here, ‘y intercept’ means the y coordinate of where the straight line graph cuts the y-axis:

(11) $$\left(0,cB1_{i,j})=(Rx_{i,j(U)}.y)-(m_{i,j})(Rx_{i,j(U)}.x\right)$$

(12) $$\left(0,cB2_{i,j})=(Rx_{i,j(L)}.y)-(m_{i,j})(Rx_{i,j(L)}.x\right)$$

where (*0,cB1_i,j_*) = the ‘y intercept’ of the first border line for the i-th transmitter and j-th Receiver while (*0,cB2_i,j_*) = the ‘y intercept’ of the second border line for the i-th transmitter and j-th receiver.

**Figure 4.**
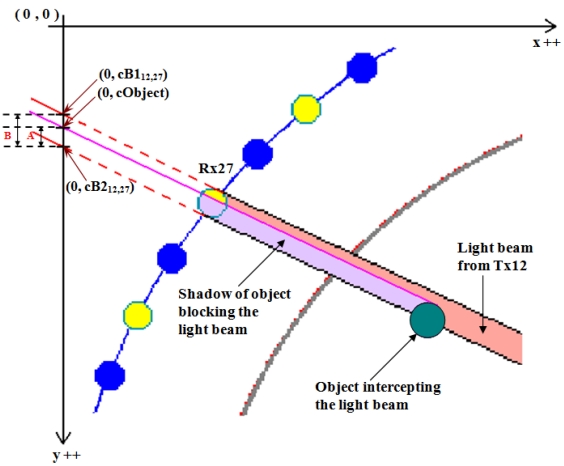
An example of an object intercepting a light beam.

Basically in modelling, an object is formed by pixels as well. Figure 4 shows an example of an object intercepting a light beam from T × 12 to R × 27. To find out the sensors condition when an object blocks the light beam, each of the pixels that forms the object has to be extrapolated to find the ‘y intercept’ of that particular pixel using all the possible combinations of gradients found in Equation 10. By using Visual C++ programming, the ‘y intercept’ value of the object labelled as (0, cObject) in Figure 4 is obtained dynamically, which means this value will vary when processed with different gradient values. If the ‘y intercept’ of the pixels that form the object lie in between the first (0, cB1_ij_) and second (0, cB2_ij_) extrapolated ‘y intercepts’ of any corresponding light paths, the ratio of the intercepting pixels to the width of the light beam is being calculated.

However, if any of the ‘y intercept’ for pixels formed by the object has the same value, this value is only counted once and the rest are ignored. ‘**A**’ in Figure 4 summarizes the shadow of object blocking the light beam while ‘**B’** is the total pixels area of the light beam as referred to when x-axis is zero as reviewed in Equations 13, 14, 15, 16 and 17.

(13) $$Z(x,y)=\{\begin{array}{cc} Z(x,y) & ;\text{if}\;\text{pixel}\;\text{colour}=\text{green}\\ 0 & ;\text{if}\;\text{pixel}\;\text{colour}\neq \text{green} \end{array}$$

(14) $$c{\text{Object}}_{i,j}=Z.y-m_{i,j}(Z.x)$$

(15) $$P_{i,j}=\{\begin{array}{ll} 1 & ;cB1_{i,j}\leq c{\text{Object}}_{i,j}\leq cB2_{i,j}\\ 0 & ;\text{otherwise} \end{array}$$

(16) $$B_{i,j}=\sum_{i=0}^{32}\;\sum_{j=0}^{6}P_{i,j}$$

(17) $$A_{i,j}=cB2_{i,j}-cB1_{i,j}$$

where:

*Z(x,y)* = all the possible coordinates of the pixels which form the object, obtained using programming looping to retrieve the colour of the pixels (drawn with green colour to represent the object). The range of x and y of *Z(x,y)* are 64 ≤ *x* < 576 and 64 ≤ *y* < 576.
*cObject_i,j_* = all the possible ‘y intercepting’ of objects for i-th transmitter and j-th receiver.
*Z.y* = the y-th coordinate of the possible object pixels obtained in Equation 13.
*Z.x* = the x-th coordinate of the possible object pixels obtained in Equation 13.
*m_i,j_* = all the gradients for i-th transmitter and j-th receivers attained in Equation 10
*p_i,j_* = an array to store the frequency of ‘y intercepting’ of objects which fulfil the given conditions where 1 is when the ‘y intercepting’ of object falls in between the border lines of the light beam for a particular i-th and j-th light beam; and it is 0 when the conditions are not fulfilled. Since the object vary in size, it is also possible that the more than one of the *Z*(*x,y*) pixels has the same *cObject_i,j_* in a single light beam. If this happens, this value is discarded or equals to 0.
*cB1_i,j_* = the ‘y intercept’ of the first border line for the i-th transmitter and j-the receiver.
*cB2_i,j_* = the ‘y intercept’ of the second border line for the i-th transmitter and j-the receiver.
*B_i,j_* = a counter to sum all ‘y intercepts’ of object pixels obtained in Equation 15 according to the i-thtransmitter and j-th receiver.
*A_i,j_* = the width of the light beam for i-th transmitter and j-th receiver when it cuts the y-axis at x-axis equals to zero.

In order to predict sensor values for various flow models, Equation 18 is applied:

(18) $$V_{i,j}=\frac{A_{i,j}}{B_{i,j}}\times V_{{\text{ref}}_{i,j}}$$

where *V_i,j_* = the predicted sensor output for i-th transmitter and j-th receiver, *V_ref_i,j__* = the assumed voltage reference for i-th transmitter and j-th receiver. As said earlier in this section, this modelling uses the tangent method to model the sensors, thus the light beams have a constant and it is equals 5 volts in this case.

## 3. Sensitivity Maps

**Figure 5.**
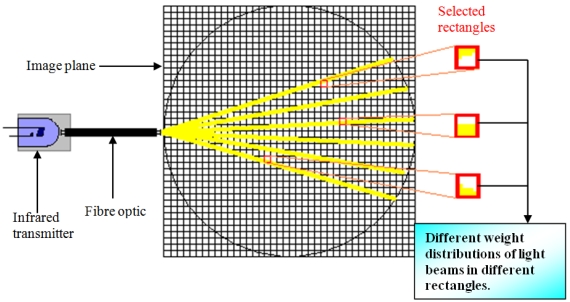
Fan beam projection map.

From the fan beam projection properties, it is known that the fan beam projection can be seen as a point source of radiation that emanates a fan shaped beam. The emanation covers many directions in different angles. For a given source and detector combination, functions are calculated that describe the sensitivity of each source and measurement pair to changes in optical properties within each pixel of the model [8]. Thus, when the projection beam is mapped onto the two-dimensional image plane, each light beam will spread across each rectangle in different weights, as illustrated in Figure 5.

The solution of the forward problem generates a series of sensitivity maps, and the inversion of these sensitivity matrixes therefore provides a reconstruction of the optical properties [8]. Basically, the number of generated sensitivity maps is dependent to the number of projections for the sensors. No matter whether the applied projection method is the 2-projection or 4-projection method, each light beam must be sampled individually. Therefore, for the thirty-two transmitters and the corresponding six receivers which receive light per emission of each transmitter, the total projections are 192. This means that there will be 192 sensitivity maps generated as well. Before these sensitivity maps can be used for image reconstruction, they must first be normalized.

Normalization of sensitivity maps is done when there is no simple linear relationship between the weight distributions in a rectangle for different light projections. For example, the rectangle (25, 30) might have certain percentage of light passing through for the light beam of transmitter 10 and receiver 25. When transmitter 0 emits light to receiver 16, this light beam might pass through rectangle (25, 30) with a different weight distribution. If there are other light beams passing through the same pixel as well, the weight distribution of the pixel will become complicated. Thus, it is necessary to build a general relationship between the pixels and all the projection beams that pass through the pixels. A simple approach to normalize the rectangle values in this research is engaged in a similar manner as normalizing the pixel values in ECT so that they have the values of zero and one when the rectangle contains the lower and higher weight distributions respectively.

**Figure 6.**
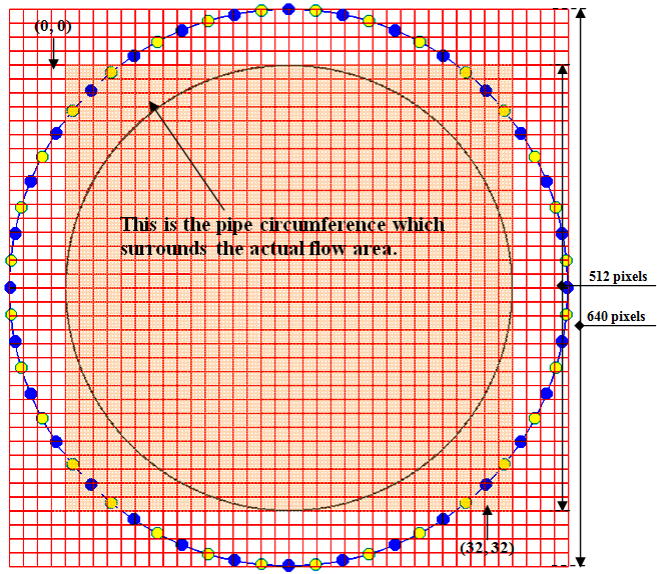
The actual flow image plane.

As a reference to map the sensitivity maps onto the 2-dimension image plane, see Figure 6. From the 640 × 640 pixels plane, there are gridlines to divide the pixels into 40 × 40 rectangles where each rectangle contains 16 × 16 pixels. In image reconstruction, the actual area of flow is from pixel 64 to pixel 575 in the x-axis and y-axis. In this section and the following section, the image plane is reconfigured so that all calculations and scanning of pixels are done only for the part of the actual flow area. This is being done to avoid wasting unnecessary time and resources used to calculate and scan the whole 640 × 640 pixels. The resolution of the actual image plane thus becomes 32 × 32 in image reconstruction which contains 512 × 512 pixels.

After the actual flow area has been identified, the sensitivity maps are generated using Visual C++ programming language, as shown in Equation 19:

(19) $$\begin{array}{cc} S_{i,j}(x,y)=\sum_{a=0}^{511}\sum_{b=0}^{511}P_{x,y}(a,b) & \left\{ \begin{array}{l} P_{x,y}(a,b)=1\;;\;\text{if}\;\text{black}=\text{changed}\\ P_{x,y}(a,b)=0\;;\;\text{if}\;\text{white}=\text{unchanged} \end{array}\right. \end{array}$$

where *S_i,j_(x, y)* = sensitivity map of light beams for all transmitters and receivers, *P_i,j_(a, b)* = an array to represent the total black pixels in rectangle-xy, *a* and *b* = the *a*-th column and *b*-th row of the pixels in the actual flow plane and the *x* and *y* = the *x*-th column and *y*-th row of the 32 × 32 rectangles or resolution.

The next step is to sum and normalize all the 192 sensitivity maps using the following two approaches:

- Summing the maps according to the weights of the same rectangles from all projections and normalizing the maps (rectangle-based normalization).
- Summing the maps based on weights of all the rectangles for individual light projection and normalizing the maps (projection-based normalization).

The rectangle-based normalization is to total up all the total black pixels from all different projections in a same rectangle using Equation 20 and normalize the maps according to Equation 21:

(20) $$\begin{array}{cc} T1(x,y)=\sum_{i=0}^{32}\sum_{j=0}^{16}S_{i,j}(x,y) & \left\{ \begin{array}{l} 0\leq x<32\\ 0\leq y<32 \end{array}\right. \end{array}$$

(21) $$N1_{i,j}(x,y)=\{\begin{array}{ll} \frac{S_{i,j}(x,y)}{T1(x,y)} & ;\text{for}\;T1(x,y)>0\\ 0 & ;\text{for}\;T1(x,y)=0 \end{array}$$

where *T*1*(x, y)* = the total or sum of the same element in rectangle-xy obtained from the 192 sensitivity maps, *S_i,j_(x, y)* = the 192 sensitivity maps attained from Equation 19 and *N*1*_i,j_(x, y)* = the normalized sensitivity maps for light beams of all Tx (0 ≤ *i* < 32) to Rx (0 ≤ *j* < 6) using the rectangle-based normalization.

Meanwhile, by using the projection-based normalization, the total weights of pixels for all rectangles (32 rectangles) for each light projection is being summed by using Equation 22 and then normalized as stated in Equation 23. The normalized maps generated in Equation 21 and Equation 23 have a total of 192 maps each:

(22) $$T2(i,j)=\sum_{x=0}^{32}\sum_{y=0}^{32}S_{i,j}(x,y)$$

(23) $$\begin{array}{cc} N2_{i,j}(x,y)=\frac{S_{i,j}(x,y)}{T2(i,j)} & \left\{ \begin{array}{l} T2(i,j)>0\\ 0\leq i<32\\ 0\leq j<6 \end{array}\right. \end{array}$$

where *T*2(*i, j*) *T*2*(i, j)* = the total of pixel weights for all rectangles in individual light projection, *S_i,j_(x, y)* = the 192 sensitivity maps attained from Equation 19 and *N*2*_i,j_(x, y)* = the normalized sensitivity maps for light beams of all Tx to Rx using the projection-based normalization.

## 4. Results

From the forward modelling, the sensor output voltage when obstructed by phantom solid objects can be predicted. From there, the inverse problem implements the image reconstruction algorithm which is able to further predict the concentration profile of the object. It is obviously desirable to have quantitative procedures for evaluating the fidelity of such tomographic images in order to assess overall system performance under various flow conditions and to compare the performance of different sensors, image reconstruction algorithms and others.

### 4.1. Multiple Objects Flow Models

**Figure 7.**
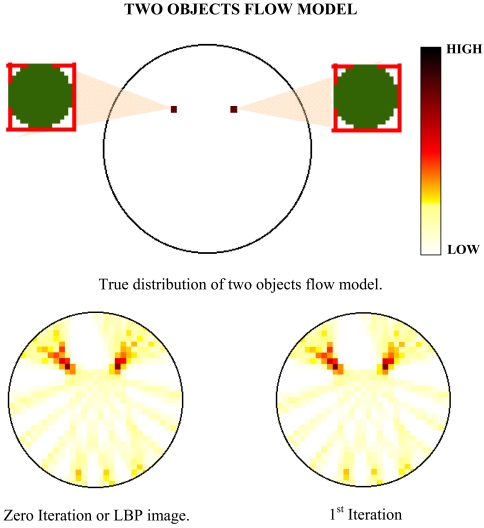

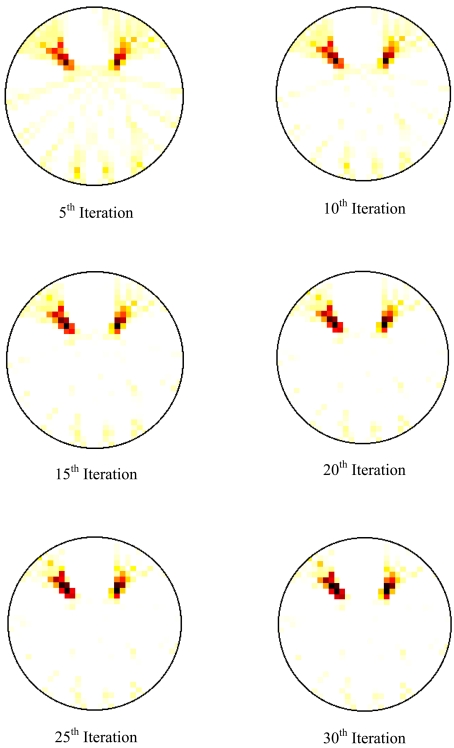
Tomogram results for two objects flow model.

**Figure 8.**
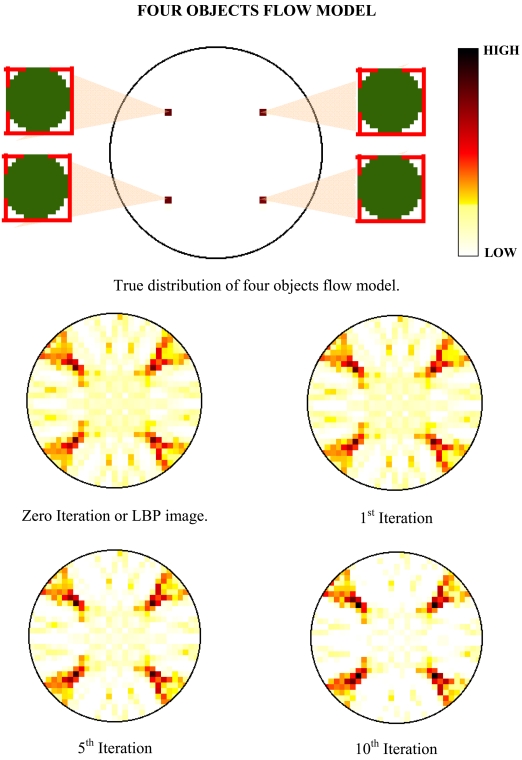

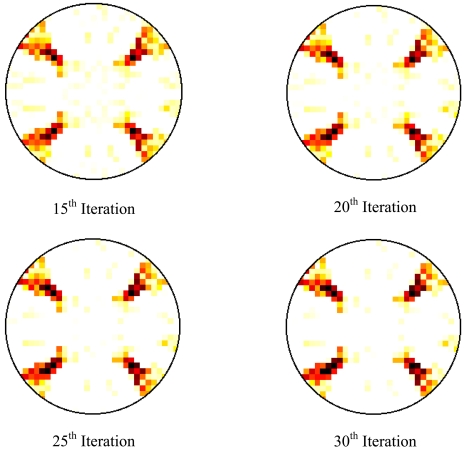
Tomogram results for four objects flow model.

The aim of investigating the multiple objects flow model is to test the aliasing effect of the reconstructed image. The implementation of optical fibre fan beam projection is proposed to eliminate the ambiguous images when two or more images are considered to intercept the sensing zone. For example, by using the LBP in parallel beam projection, four images are detected in the tomogram but in reality, only two objects are intercepting the sensing zone. The other two images are known as the ambiguous or false images that contribute to the huge error and confusion. In this experiment to test the aliasing effect of the fan beam projection, two models are proposed which are the two objects and four objects flow models. All the objects have a diameter of 2.50 mm each and the reconstructed images are being assessed using the Spatial Image Error (SIE), Peak Signal-to-Noise Ratio (PSNR) and Mean Square Error (MSE) error analyses. The tomograms which are obtained from the experiments are shown in Figures 7 and 8.

The calculations of SIE, MSE and PSNR for the two objects and four objects flow model are listed in Table 1 below.

**Figure 9.**
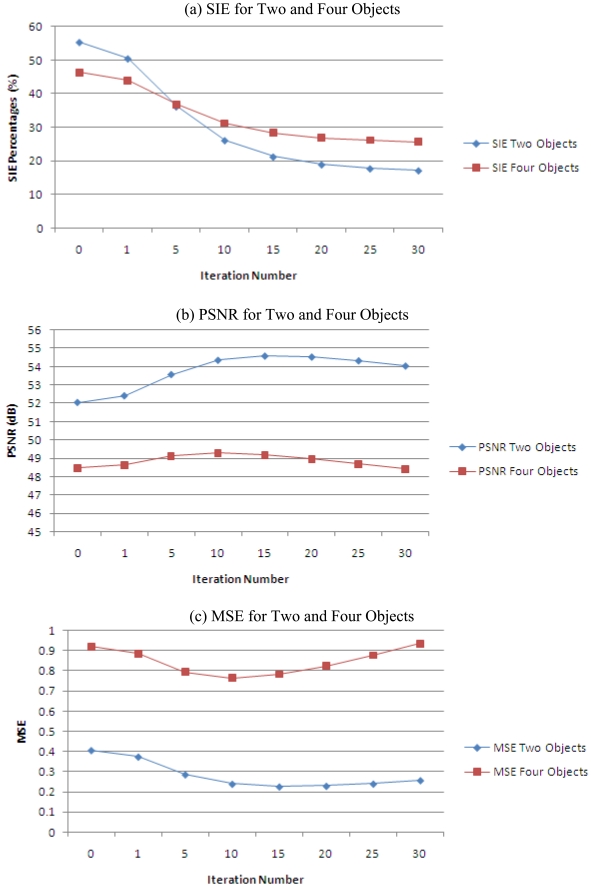
SIE, PSNR and MSE error analyses.

The SIE, PSNR and MSE error data for both flow models can be presented in a clearer manner by plotting the values in line graphs as shown in Figure 9.

Generally, from the results of the tomogram, it is found out that the optical fibre fan beam projection does not have ambiguous images for multiple objects flow model. Samilarly to the previous conclusion, the LBP or at zero iteration, the multiple objects models yields the highest SIE error which means that the images produce many smearing. When applied with the iterative reconstruction algorithm, the optimized iteration level is also observed at the 10^th^ iteration. In addition, the percentage of improvement of the 10^th^ iteration when compared to the LBP image for both the images is also being calculated. The results of the calculations are shown in Table 2 and the average improvement of the iterative algorithm at 10^th^ iteration for multiple objects flow is 42.74%.

## 5. Discussion

From the error assessment theory that has been discussed earlier, it is known that a low SIE and MSE but with a high PSNR represent a better image quality. The analyses of the graphs show that basically, the LBP algorithm does not contribute to a good image quality because the images reconstructed using LBP is often distorted [9] and blurred. The iterative reconstruction algorithm offers a solution to minimize the blurring artifacts of reconstructed image caused by using the LBP algorithm. This advantage can be clearly observed for the multiple objects flow model.

However, there is a maximum limit to apply the number of iterations that will improve the images (by observing the maximum point of the PSNR and the point where the SIE and MSE error measurement starts to become static or even decreases in a few cases). This point is also referred to as the optimized level to perform image reconstruction. After this point, while the PSNR continues to decrease, the SIE does not appear to reduce rapidly. Thus, the further iterations after the optimized level will only become the disadvantages by lowering the PSNR and also stretching the image processing time without giving significant improvements to the image quality.

Overall, the resolution of the sensors is quite good since it can give a reliable measurement of solid objects as small as 0.45 mm. Besides that, the optical fibre sensors are able to obtain the image of multiple objects without ambiguity. The iterative reconstruction algorithm has the potential to minimize the smearing around the image that is clearly present in the image generated using LBP algorithm.

## Figures and Tables

**Table 1.** Assessment of reconstructed images for two and four objects flow model.

**Iteration Number**	**SIE**	**Two Objects MSE**	**PSNR**	**SIE**	**Four Objects MSE**	**PSNR**
0	55.193	0.406	52.044	46.381	0.921	48.488
1	50.33	0.374	52.408	44.011	0.887	48.651
5	36.084	0.286	53.57	36.785	0.796	49.122
10	26.048	0.239	54.351	31.228	0.765	49.292
15	21.178	0.226	54.581	28.419	0.783	49.196
20	18.878	0.229	54.528	26.957	0.825	48.968
25	17.69	0.24	54.321	26.119	0.878	48.694
30	17.099	0.257	54.036	25.644	0.937	48.412

**Table 2.** Improvement of reconstructed image using SIE error analysis.

**Flow Model**	**0th Iteration or LBP**	**10th Iteration**	**Improvement**
Two objects	55.193	26.048	52.81%
Four objects	46.381	31.228	32.67%
Average Improvement			42.74%
